# Naïve CD4^+^ T Cell Activation in the Nasal-Associated Lymphoid Tissue following Intranasal Immunization with a Flagellin-Based Subunit Vaccine

**DOI:** 10.3390/ijms232415572

**Published:** 2022-12-08

**Authors:** John T. Bates

**Affiliations:** 1 Department of Microbiology & Immunology, Wake Forest School of Medicine, Winston-Salem, NC 27157, USA; jtbates@umc.edu; 2 Department of Cell & Molecular Biology, University of Mississippi Medical Center, Jackson, MS 39216, USA; 3 Department of Medicine, University of Mississippi Medical Center, Jackson, MS 39216, USA; 4 Center for Immunology and Microbial Research, University of Mississippi Medical Center, Jackson, MS 39216, USA

**Keywords:** nasal-associated lymphoid tissue, flagellin, CD4^+^ T cells, immune induction, respiratory immunity

## Abstract

The nasal-associated lymphoid tissues (NALT) are generally accepted as an immune induction site, but the activation of naïve T-cells in that compartment has not been well-characterized. I wanted to determine if early events in naïve CD4^+^ T cell activation and the extent of antigen specific cell division are similar in NALT to that observed in other secondary lymphoid compartments. I performed antigen tracking experiments and analyzed the activation of naïve antigen-specific CD4^+^ T cells in the nasal-associated lymphoid tissues (NALT). I directly observed transepithelial transport of fluorescently labeled antigen from the lumen of the airway to the interior of the NALT two hours following immunization. One day following intranasal (i.n.) immunization with antigen and adjuvant, antigen-specific CD4^+^ T cells in the NALT associated as clusters, while antigen-specific CD4^+^ T cells in control mice immunized with adjuvant only remained dispersed. The antigen-specific CD4^+^ populations in the NALT and cranial deep cervical lymph nodes of immunized mice expanded significantly by day three following immunization. These findings are consistent with initial activation of naïve CD4^+^ T cells in the NALT and offer insight into adjuvant mechanism of flagellin in the upper respiratory compartment.

## 1. Introduction

The NALT is generally considered an immune inductive site [1,2,3,4], though numerous questions about the role of NALT following intranasal (i.n.) immunization remain [5]. The compartment in mice is small and easily destroyed during harvest. Consequently, few data are available on the in situ activation of immune cells in the NALT following i.n. immunization. Previous reports have shown that i.n. immunization with experimental subunit vaccines results in an increase in the size of the lymph nodes that drain the upper respiratory tract [6], but detecting changes in the lymphoid microenvironments and composition of the NALT requires multiple immunizations [7] and has not been demonstrated at an early time point following a single immunization.

Given these observations, I wanted to determine if the immune response to primary i.n. immunization with a subunit vaccine is initiated in the NALT. Bacterial flagellin, the ligand for TLR5 [8] and NLRC4 [9,10], was used as an adjuvant for these studies. Flagellin is a potent vaccine adjuvant [11,12] that has been shown to be safe in Phase I trials [13,14,15]. Intranasal immunizations that include flagellin as an adjuvant are effective at stimulating mucosal immunity against a wide range of pathogens [6,7,16,17,18,19,20,21,22,23,24,25,26,27,28,29]. Because flagellin is a protein, in many experimental systems it has been possible to create a single molecule vaccine comprising both adjuvant and antigen [11]. This aspect of the flagellin vaccine platform offers two very significant strengths. The first is that the antigen-specific T cell response to a fusion protein is significantly greater than the response to flagellin and antigen administered as separate proteins [30,31], likely as a result of delivering antigen and adjuvant stimulation to the same antigen-presenting cells. Second, production of a vaccine consisting of a single molecule streamlines pre-clinical development and production of the final product. I used an ovalbumin-specific CD4^+^ T cell adoptive transfer model system to directly visualize early cellular events in the NALT following a single i.n. immunization with flagellin-ovalbumin fusion protein.

## 2. Results

### 2.1. Luminal Sampling of Antigen by NALT Cells

**Figure 1:**
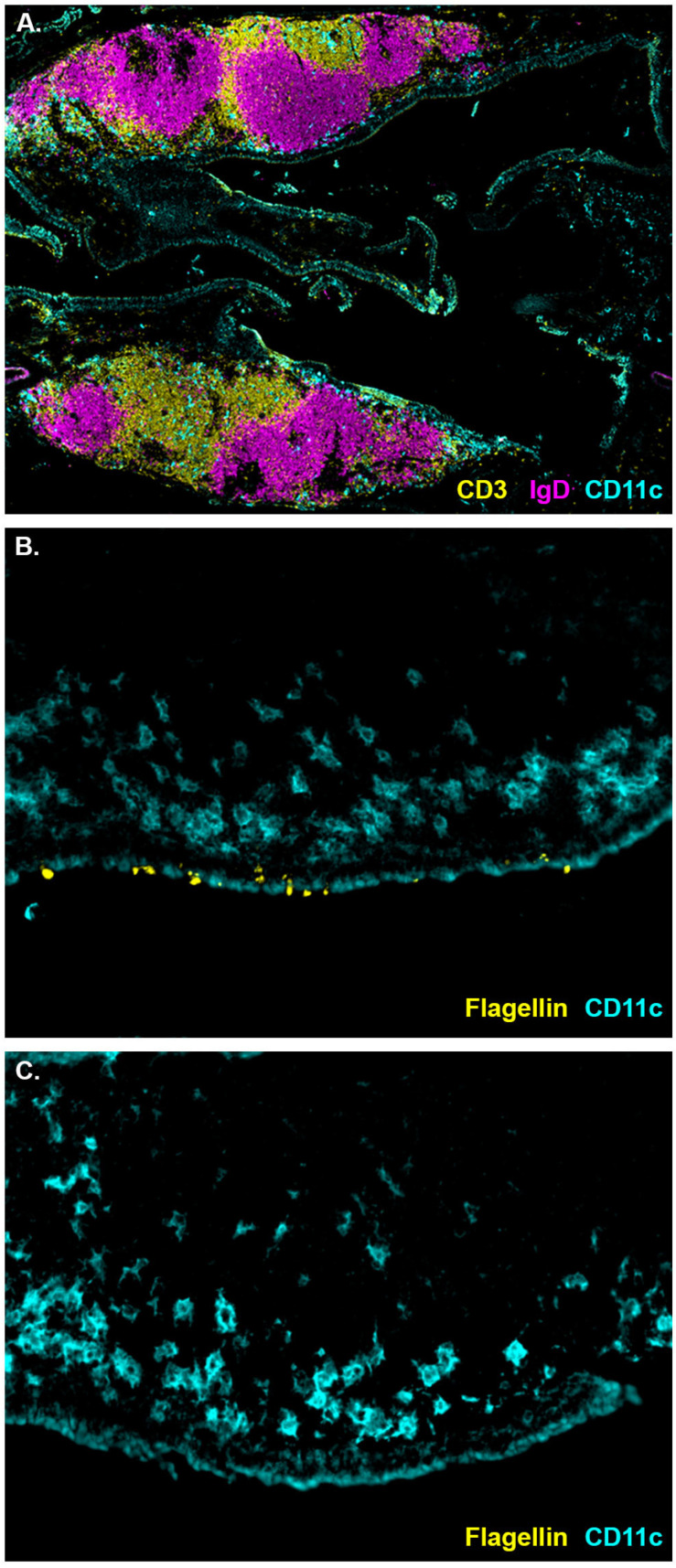
Cell populations and antigen sampling in the NALT of naïve mice. (**A**) Both NALT from one mouse showing IgD^+^ cells (magenta), CD3^+^ cells (yellow), and CD11c^+^ cells (cyan) (N = 4). (**B**) Luminal surface of the NALT bound by fluorescently labeled flagellin (yellow) two hours following i.n. instillation with CD11c^+^ cells (cyan) in the subepithelial region (N = 4). (**C**) Luminal surface of the NALT two hours following i.n. instillation of fluorescently labeled flagellin (yellow) mixed with an excess of unlabelled flagellin with CD11c^+^ cells (cyan) in the subepithelial region (N = 4).

The NALT are situated above the soft pallet, and lymphocytes within the NALT are segregated into T and B cell zones (Figure 1A). The epithelium covering the NALT comprises goblet cells, epithelial cells, and M cells [7,32]. While the NALT are generally considered to be an immune induction sight, direct visualization of initiation of naïve immune responses in the NALT is lacking. Similarly, attempts to visualize sampling of flagellin by the NALT have been unsuccessful [7]. To determine if cells covering the NALT sample flagellin from the lumen of the airway, I i.n. instilled C57BL/6J mice with seven μg of fluorescently labeled flagellin. A second group of mice received labeled flagellin and an additional 14 μg of unlabelled flagellin to act as a competitor. Two hours later the mice were euthanized and the NALT removed. This time point was chosen with the presumption that it would allow for mucociliary clearance of unbound flagellin while still being early enough to see labeled flagellin prior to antigen processing. Labeled flagellin was observed bound to the luminal surface of the NALT and was also visible on the abluminal side of the epithelium (Figure 1B). However, labeled flagellin was not detectable in mice that received labeled flagellin mixed with an excess of unlabeled flagellin (Figure 1C). Six hours following instillation, labeled flagellin was no longer detectable. The staining pattern of flagellin appears consistent with active transport of flagellin across the epithelium. This transport is likely mediated by dendritic cells or NALT M cells. Both cell types are present in the NALT epithelium of mice [7,33] and are known to be involved in antigen sampling in other mucosal compartments [34]. Notably, internalized flagellin was detected in close proximity to a CD11c^+^ cell population near the epithelium (Figure 1B). This population of dendritic cells (DC) lies along the edge of the B cell zone and may be equivalent to the subepithelial dome DC population in the Peyer’s Patch. In the Peyer’s Patch, IgA production requires B cell interaction with subepithelial dendritic cells [35], and this population the NALT may be crucial to the IgA response following i.n. immunization with flagellin. Alternatively, antigen sampling in the NALT may be mediated by a population of CD103+ DC similar to those that perform transepithelial sampling of bacterial antigens in the gut [36].

### 2.2. Clustering of Naïve CD4^+^ T Cells in the NALT following Intranasal Immunization

**Figure 2:**
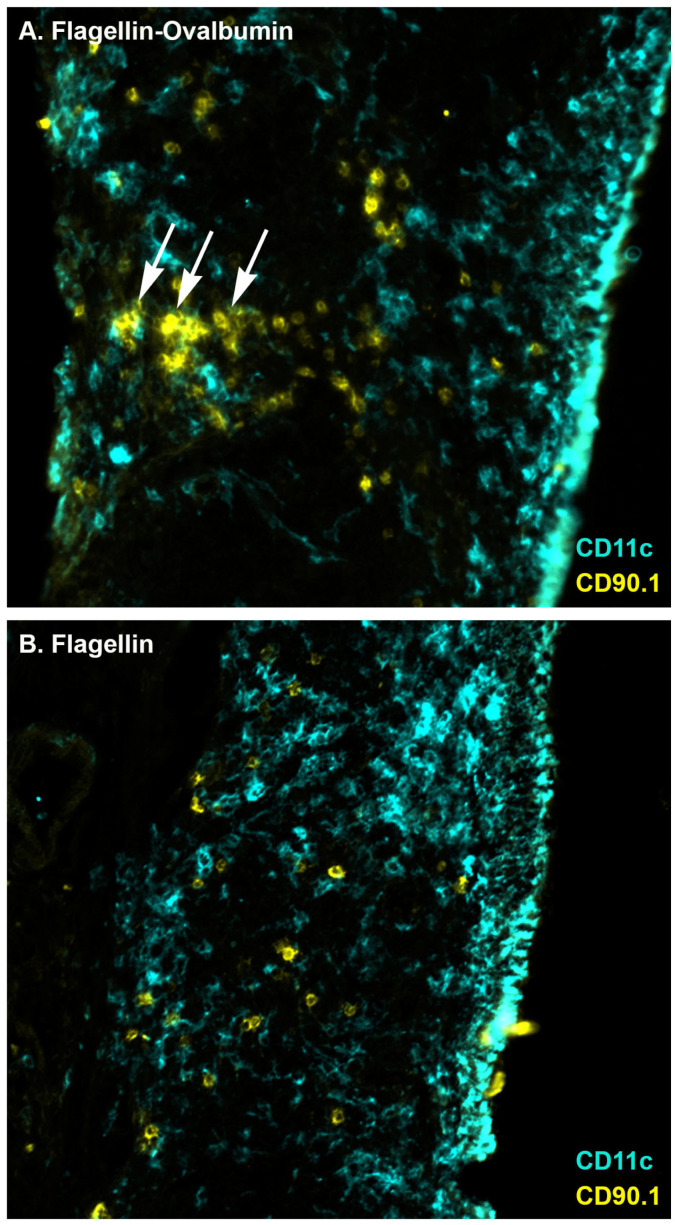
Clustering of OVA-specific CD4^+^ T cells in the NALT following immunization with flagellin-OVA fusion protein. Mice were i.n. immunized with (**A**) flagellin-ovalbumin (N = 6) or (**B**) flagellin (N = 3). The following day, mice were euthanized, and NALT were harvested. Tissues were frozen, sectioned, and stained to reveal OVA-specific CD90.1^+^ T cells (yellow) and CD11c+ cells (cyan). Arrows indicate clusters of OVA-specific CD4^+^ T cells.

Clustering of naïve CD4^+^ T cells around dendritic cells has been used as a measure of antigen presentation to and early activation of CD4^+^ T cells in draining lymph nodes [31,37]. To determine if CD4^+^ T cells in the NALT exhibit the same behavior, I performed immunization studies in wild type C57BL/6J mice that received adoptive transfer of 5 × 10^6^ OVA_323-339_-specific OT-II cells. Mice were immunized one day following cell transfer, and NALT were harvested the following day. OVA-specific cells in the NALT of mice immunized with flagellin-OVA fusion protein were present in clusters (Figure 2A). Notably, OVA-specific cell clusters did not occur around CD11c^+^ cells near the epithelium but in proximity to CD11c^+^ cells located farther from the luminal surface of the NALT, which is consistent with transport of antigen from the subepithelial region of the NALT to the T cell region of the NALT. OVA-specific cells in the NALT of control mice immunized with only flagellin remained dispersed (Figure 2B), demonstrating dependence of CD4^+^ T cell activation on the presence of cognate antigen. This observation is consistent with initial activation of naïve CD4^+^ T cells in the NALT. 

### 2.3. Proliferation of Naïve CD4^+^ T Cell in the NALT following Intranasal Immunization

**Figure 3:**
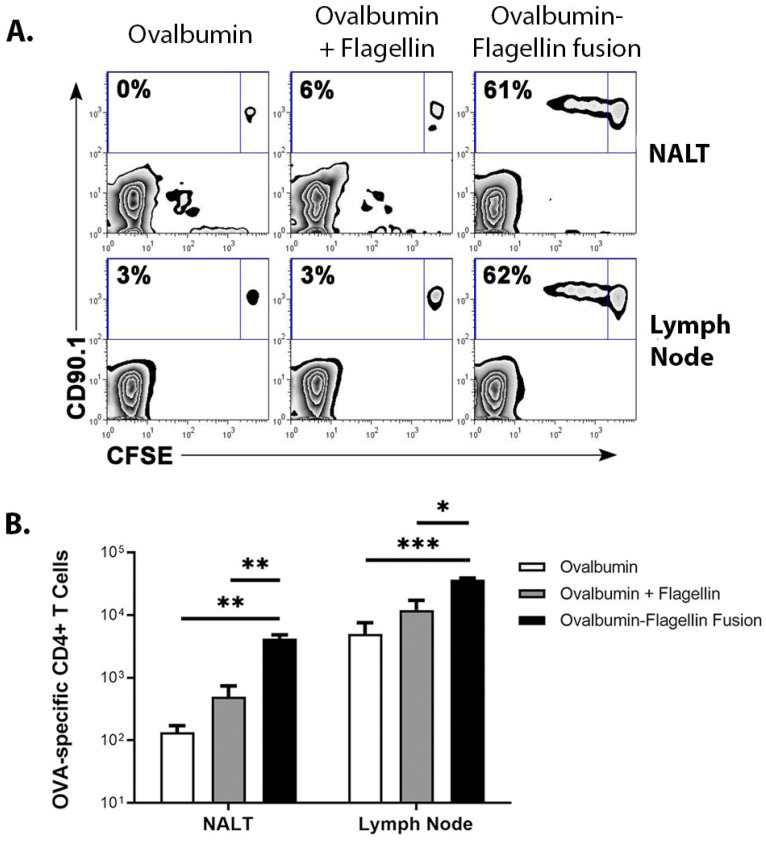
Proliferation of OVA-specific CD4^+^ T cells in the NALT following immunization with flagellin-OVA fusion protein. NALT were harvested three days following i.n. immunization with ovalbumin, flagellin plus ovalbumin, or flagellin-ovalbumin fusion protein. (**A**) CFSE dilution by OVA-specific CD4^+^ T cells was measured by flow cytometry. Plots are gated on CD4^+^ lymphocytes. Numbers convey the percentage of OVA-specific cells in each condition that underwent at least one division prior to harvest (**B**) Intranasal instillation of flagellin-ovalbumin stimulated significantly higher levels of antigen-specific cell population expansion in the NALT (*p* < 0.003) and LN (*p* < 0.001) relative to mice immunized with ovalbumin. The absolute numbers of OVA-specific CD4^+^ T cells recovered from the NALT (** *p* = 0.003) and LN (*** *p* < 0.001) of mice immunized with flagellin-ovalbumin was significantly higher than the number of cells recovered from mice immunized with ovalbumin or ovalbumin plus flagellin as separate proteins (*p* < 0.01 for NALT and *p* < 0.05 for LN). An average of 140 and 5100 CD90.1^+^ cells were recovered from the NALT and LN, respectively, of mice immunized with ovalbumin only. * denote the level of significant difference are appear lower in the figure legend. An average of 4200 and 37,000 CD90.1^+^ cells were recovered from the NALT and LN, respectively, of mice immunized with flagellin-ovalbumin. Results shown were generated from one experiment with three mice per group and representative of a total of six mice per condition across two independent experiments.

To determine if antigen-specific CD4^+^ T cell proliferation followed cluster formation, I adoptively transferred 3 × 10^6^ CFSE-labeled OVA-specific CD4^+^ T cells into C57BL/6J mice and i.n. immunized the following day with ovalbumin or flagellin-OVA fusion protein. Three days after immunization, mice were euthanized and CFSE-dilution was measured in OVA-specific CD4^+^ T cells recovered from the NALT and the cranial deep cervical lymph nodes. The three-day time point was chosen because T cell proliferation is typically well under way by this time, but the CFSE label is still present at high enough levels for analysis. This lymph node was previously identified as the most responsive node following i.n. immunization of mice [6]. OVA-specific cells recovered from the NALT and LN exhibited similar levels of cell division following i.n. immunization with flagellin-OVA. However, cells recovered from mice that were immunized with ovalbumin only or with ovalbumin and flagellin administered as separate proteins did not divide (Figure 3A). This result demonstrates the adjuvant requirement for CD4^+^ T cell activation and the increased adjuvant effect of flagellin-antigen fusion proteins compared to simultaneous administration of separate proteins. Significantly more OVA-specific CD4^+^ T cells were recovered from the NALT and cranial deep cervical LN of mice immunized with flagellin-ovalbumin than from mice immunized with ovalbumin only or with ovalbumin and flagellin administered as separate proteins (Figure 3B). Immunization with flagellin plus ovalbumin did not yield a significant increase in the number of OVA-specific T cells in the NALT compared to immunization with just ovalbumin (Figure 3B). These data confirm that the NALT is an immune induction site for the naive antigen-specific CD4^+^ T cell response following i.n. immunization and suggest that induction of the adaptive immune response depends on closely coordinated activation of innate immunity.

## 3. Discussion

The SARS-CoV-2 pandemic has highlighted our need for a better understanding of the initiation and durability of mucosal immunity. While the NALT has generally been considered an immune inductive site [1,2,3,4], findings that directly demonstrate activation of naïve CD4^+^ T cells in this compartment are limited. The NALT has been reported to support recall but not priming of CD8^+^ T cells [38]. Antigen-presenting cell populations in the NALT of mice express markers typically associated with tolerogenicity and low levels of CD86 and IAd [39]. T cells recovered from the NALT predominantly express low levels of CD45RB which is consistent with an effector or memory population [39]. Park and colleagues showed cellular division of naïve CD4^+^ T cells in the NALT using an OVA-specific adoptive transfer model system and bacterial i.n. infection [40], though their observations were made three days following infection and do not strictly demonstrate in situ activation in the NALT.

I have observed that adoptively transferred naïve CD4^+^ T cells recirculate to the NALT under steady state conditions and are activated there following i.n. immunization (Figure 2 and Figure 3). The OVA-specific CD4^+^ T cell clusters in the NALT are similar to those statically observed in draining non-mucosal lymph nodes following parenteral immunization [31,37]. Importantly, clusters in both the LN and the NALT are present one day following immunization, which suggests that initial activation of naïve T cells in both compartments is similar. Intravital microscopy studies have shown that these clusters consist of antigen-specific T cells that stop after encountering antigen-loaded DC [41] and that regulatory CD4^+^ T cells inhibit stable cluster formation [42]. Cluster formation in the NALT was followed by a significant expansion in the OVA-specific CD4+ T cell populations in the NALT and cranial deep cervical lymph nodes (Figure 2). Consequently, antigen-specific CD4^+^ T cell cluster formation in the NALT following immunization with flagellin-OVA fusion protein is consistent with the initiation of an immune response and a flagellin-mediated adjuvant effect.

These results raise some questions about possible compartmental differences in the immune response to flagellin. Flagellin’s potent adjuvant effect in the upper respiratory tract contrasts with the immunoregulatory role of TLR5-signaling in the gut. Under normal conditions, flagellin present in the gut does not elicit a strong effector immune response, though basolateral exposure of the gut mucosa to flagellin evokes a response [43]. Rather, as evidenced by the tendency of TLR5 knockout mice to develop colitis [44], flagellin contributes to negatively regulating the response to gut microbiota via IL-10 production [45,46] and by stimulating regulatory CD4^+^ T cells [47]. Flagellin’s ability to promote a strong adaptive effector immune response in the context of i.n. administered vaccines while simultaneously stimulating a regulatory response in the healthy gut is likely based on differences in the antigen presenting cell populations and deserving of further study.

The SARS-CoV-2 pandemic has highlighted the difficulty in producing a vaccine that stimulates durable immunity in the upper respiratory compartment. Development of flagellin as a vaccine platform to deliver antigen and adjuvant to the same APC in the respiratory mucosa could potentially maximize the response of antigen-specific mucosal lymphocytes, thereby generating longer-lived, vaccine-mediated respiratory immunity.

## 4. Materials and Methods

### 4.1. Immunogens

Recombinant his-tagged *Salmonella* FliC (flagellin) and his-tagged flagellin-OVA fusion proteins were produced as previously described [31,48,49]. Nucleic acids and endotoxins were removed via passage of purified protein through Acrodisc Mustang Q and E membranes (Pall). Contaminating endotoxin levels were verified to be <30 pg LPS/μg protein by *Limulus amebocyte* lysate assay (Associates of Cape Cod, East Falmouth, MA, USA). The TLR5-mediated bioactivity of flagellin and flagellin-OVA were verified by measuring TNF-α production in cultures of RAW424 cells stably transfected to express mouse TLR5 [50]. OVA was purchased from Sigma-Aldrich and passed through Mustang Q and E membranes prior to use.

### 4.2. Adoptive Transfer of OVA-Specific TCR Transgenic T Cells

OVA_323-339_-specific CD4^+^ T cells were harvested from the spleens and lymph nodes of TCR transgenic OT-II mice [51] expressing the CD90.1 molecule and were injected into congenic wild type C57BL/6J mice via the tail vein. Recipient mice in the cell clustering experiments received 5 × 10^6^ OVA-specific CD4^+^ T donor cells, and recipient mice in the carboxyfluorescein succinimidyl ester (CFSE) experiments received 3 × 10^6^ donor cells. Mice were i.n. immunized with 10^−10^ moles of immunogen (e.g., 8 µg of flagellin-OVA fusion protein) in a total volume of 15 µL of PBS one to two days following cell transfer. Mice were euthanized by CO_2_ asphyxiation at the stated times. OT-II and CD90.1 breeder mice and C57BL/6 mice were purchased from The Jackson Laboratory and housed in an AAALAC-approved barrier facility. All animal experiments were conducted in accordance with institutional and NIH guidelines and approved by the IACUC at Wake Forest University School of Medicine.

### 4.3. Immunofluorescence

NALT were harvested and frozen in Optimal Cutting Temperature (OCT) compound (Sakura Finetek USA, Torrance, CA, USA). Frozen sections that were six microns thick were cut from tissue blocks and dehydrated in acetone. CD3^+^ cells were identified by staining with 145-2C11 conjugated to FITC (BD Bioscience, Franklin Lakes, NJ, USA) followed by rabbit anti-FITC polyclonal antibody conjugated to AlexaFluor488 (Invitrogen, Waltham, MA, USA). IgD^+^ cells were identified by staining with unlabeled 11-26c (eBioscience, San Diego, CA, USA) followed by donkey anti-rat IgG conjugated to AlexaFluor647 (Invitrogen). CD11c^+^ cells were revealed by staining with biotinylated HL3 (BD Biosciences) followed with streptavidin conjugated to AlexaFluor594 (Invitrogen).

For antigen tracking experiments, recombinant flagellin was conjugated to AlexaFluor647 using a protein labeling kit purchased from Invitrogen. NALT were harvested and fixed in 3% paraformaldehyde prior to freezing in OCT. Fluorescently labeled flagellin was directly visualized. CD11c^+^ cells were visualized by staining with HL3 conjugated to biotin (BD Biosciences) followed by streptavidin conjugated to AlexaFluor568 (Invitrogen).

For adoptive transfer experiments, TCR transgenic OT-II cells were identified on the basis of CD90.1 expression using the CD90.1-specific monoclonal OX-7 conjugated to FITC (BD Biosciences) followed by rabbit anti-FITC^AF594^ (Invitrogen). CD11c+ cells were identified by staining with biotinylated N418 (Biolegend, San Diego, CA, USA) followed by streptavidin conjugated to AlexaFluor568. 

All tissue staining was performed at room temperature. Slides were incubated for 30 min with blocking buffer (1% bovine serum albumin in PBS), and antibodies were incubated on slides for approximately 30 min before washing with blocking buffer. Slides were washed 3× at each wash step with a volume of ~2 mL of washing buffer dispensed by a disposable transfer pipette. Cover slips were applied using ProLong Gold antifade mounting medium (Invitrogen). Slides were imaged using a Nikon Eclipse TE300 microscope and a Retiga EX camera. Adjustments in brightness, contrast, and levels were applied to raw images, and color composite images were composed using Adobe PhotoShop CS6.

### 4.4. CFSE Labeling and Flow Cytometry

CFSE labeling was performed by incubating 2.5 × 106 cells/mL serum-free PBS containing 2 μM CFSE (Invitrogen) for 10 min at room temperature. Adoptively transferred OT-II cells were discriminated on the basis of binding by CD90.1-specific monoclonal OX-7 (BD Biosciences, cat. # 557266) and CD4-specific monoclonal RM4-5 (BD Biosciences, cat # 557681). Data were collected using a FACSCalibur flow cytometer (Becton Dickinson) and analyzed with FloJo 7.2.5 (Tree Star). Absolute cell numbers were determined by flow cytometric bead-based counting [52]. Approximately 32,000 lymphocyte events were acquired from samples from mice treated with ovalbumin and approximately 100,000 events were acquired from samples from mice treated with flagellin-ovalbumin.

## Data Availability

Data is contained within the article.
